# FGF21 deletion mildly exacerbates hepatic dysfunction in GAN diet and alcohol fed rats

**DOI:** 10.1038/s44324-025-00062-5

**Published:** 2025-05-28

**Authors:** Peter Aldiss, Malte Hasle Nielsen, Hayley Burm, Denise Oró, Henrik H. Hansen, Michael Feigh, Matthew P. Gillum

**Affiliations:** 1 Nottingham Digestive Diseases Centre, Translational Medical Sciences, and the Biodiscovery Institute, University of Nottingham, Nottingham, NG7 2RD UK; 2 National Institute of Health Research (NIHR) Nottingham Biomedical Research Centre, Nottingham University Hospitals NHS Trust, and University of Nottingham, Nottingham, UK; 3 Novo Nordisk Foundation Center for Basic Metabolic Research, Faculty of Health and Medical Sciences, University of Copenhagen, 2200 Copenhagen, Denmark; 4 Gubra, Horsholm, Denmark; 5 Department of Biomedical Sciences, Faculty of Health and Medical Sciences, University of Copenhagen, 2200 Copenhagen, Denmark

**Keywords:** Endocrinology, Endocrine system and metabolic diseases, Liver, Liver diseases, Metabolism

## Abstract

Fibroblast growth factor 21 (FGF21) analogues are in clinical development as treatments for metabolic and alcohol-associated liver disease. The aim of this study was to characterize the first FGF21 knockout (KO) rat line to validate its utility as a translational animal model that recapitulates human disease. We generated an FGF21 KO rat model and exposed 6-month-old WT and KO rats to either chow (*n* = 8 per genotype) or the obesogenic GAN (Gubra Amylin NASH) diet (*n* = 16 per genotype) for 12 weeks. Lack of endogenous FGF21 increased plasma transaminases, liver weight, and total levels of liver TG in GAN-fed FGF21 KO rats. FGF21 KO had no impact on body weight, glycaemic traits, or MASH histological endpoints, including hepatic steatosis, NAS score, lobular inflammation, ballooning degeneration, fibrosis stage, or the liver transcriptome. Finally, we demonstrate that endogenous FGF21 does not regulate drinking behaviour in rats.

## Introduction

Obesity and type 2 diabetes are associated with multiple co-morbidities, including excess lipid accumulation in the liver and the progression to metabolic dysfunction-associated steatotic liver disease (MASLD)^1^. MASLD is the most common chronic liver disease in the Western world and is a major risk factor for hepatocellular carcinoma and cirrhosis^1^. During this progression, MASLD advances to metabolic dysfunction-associated steatohepatitis (MASH), where the liver is characterised by lobular inflammation, steatosis, and ‘ballooning’, or hepatocyte injury^1^. At present, there are no FDA-approved treatments for MASH, and clinically relevant models are sparse; as such, there is a critical need for the development of these models to support drug-development pipelines in the search for effective treatments.

Fibroblast growth factor 21 (FGF21) is a liver-secreted hormone, or hepatokine, which is also expressed in adipose tissues. Originally identified as a ‘starvation’ factor, it is now clear that FGF21 has numerous physiological roles, including the regulation of glucose and lipid metabolism, energy balance, and appetite, which are mediated through its binding to FGF receptor 1 (FGFR1) and the co-receptor β-klotho (KLB).

Mechanistically, FGF21 is a critical regulator of hepatic metabolism and plays a key role in the development of MASLD^2^. FGF21 knockout mice develop significant hepatic steatosis in response to ketogenic, and amino acid-deprived diets through impaired fatty acid oxidation and increased lipogenesis^3–5^. More broadly, FGF21 knockout mice are hyperinsulinaemic and develop attenuated liver insulin sensitivity in response to an obesogenic diet^6,7^. Conversely, FGF21 overexpression, or treatment with FGF21, improves insulin sensitivity, reduces hepatic steatosis, increases thermogenic capacity of white and brown adipose tissues, and improves β-cell function^2^. Further, FGF21 analogues consistently reverse or reduce MASH in genetic, diet, and chemically induced pre-clinical models, demonstrating the therapeutic potential of FGF21 analogues in resolving liver pathologies^8^.

In humans, circulating FGF21 is increased in MASLD and MASH, increasing with the degree of fibrosis, and is an independent predictor of hepatic steatosis^9^. Circulating FGF21 is inversely correlated with insulin sensitivity and correlates positively with visceral fat mass and circulating ALT^9,10^. Further, long-acting FGF21 analogues, which are currently in clinical trials, consistently report reductions in circulating triglycerides and cholesterol, hepatic triglycerides, and potentially improve liver fibrosis^11,12^. Despite this, the mechanisms underlying the role of FGF21 in MASH remain unclear.

Further, FGF21 is implicated in the genetics of alcohol intake and alcohol-use disorder. Pre-clinical models demonstrate that FGF21 treatment can reduce alcohol preference and intake in mice and non-human primates, where it acts through an amygdalo-striatal circuit^13^. As such, advanced models are now required to further understand FGF21 biology and its role in complex behaviours such as addiction.

To this end, and taking advantage of recent advancements in CRISPR/Cas9 technology, we capitalised on the translational value of rat models of liver disease and addiction to develop a novel, FGF21 knockout rat and determine its suitability as a genetic, physiological model for human MASH and alcohol-use disorder.

## Methods

### Ethics statement

**Fig. 1:**
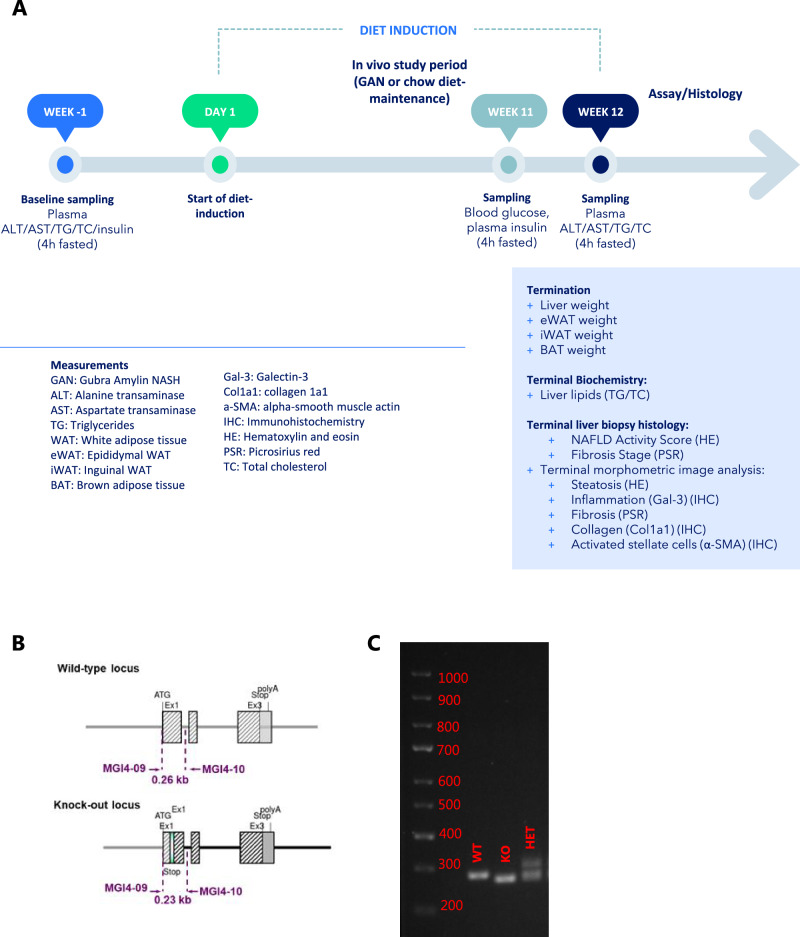
Experimental design and model validation. Overview of study design and endpoints (**A**); overview of model generation by genOway (Lyon, France) using CRISPR/Cas9 to insert an artificial STOP codon in exon 1 (**B**), and example of genotyping (**C**).

All in vivo experiments were conducted at the University of Copenhagen, Denmark, under approval from the Danish Animal Experiments Inspectorate, Danish Ministry of Food, Agriculture and Fisheries (Permit #). Forty-eight male rats (*n* = 24 WT, *n* = 24 KO) were randomised to either chow (*n* = 8 per group, 3.22 kcal/g, Altromin 1324, Brogaarden, Hoersholm, Denmark) or the Gubra-Amylin MASH (GAN) diet (*n* = 16 per group, 4.49 kcal/g, 40 kcal-% fat (of these 46% saturated fatty acids by weight), 22% fructose, 10% sucrose, 2% cholesterol; D09100310, Research Diets) for a period of 12 weeks (see Fig. 1A for study outline). Rats were pair-housed, supplied with enrichment in a temperature-controlled environment (21–23 °C) and maintained on a 12-h light-dark cycle (6:00 am–6:00 pm) with ad libitum access to food and drinking water, unless otherwise specified. Animals were fasted for 4 h in their home cage before being terminated by cardiac puncture under isoflurane anaesthesia.

For alcohol studies, a separate cohort of WT and KO rats (*n* = 15 per group) was exposed to 10% ethanol for 3 weeks across 3 different drinking paradigms. In week 1, animals were given continuous access to water or 10% ethanol (two-bottle preference). In week 2, animals were exposed to the first a binge-drinking paradigm and given continuous access to water with 10% ethanol for 24 h on Mon, Wed, Fri (intermittent access two-bottle choice) In week 3, animals were exposed to a final binge-drinking paradigm where they have continuous access to water and 10% ethanol was available for 2 h periods, beginning 2 h after the onset of the dark cycle when drinking behaviour peaks (drinking in the dark). Animals were terminated by cardiac puncture under isoflurane anaesthesia.

### Model generation

The FGF21 knockout rat model was developed by genOway (Lyon, France) on a Wistar background. To generate the Knock-out, a CRISPR sgRNA targeting exon 1 of the *GFG21* gene was designed as well as a template ssODN, aiming at deleting aa29-37, the 8 amino acids critical for FGF21 activity, and replacing them with a STOP codon. Fertilised oocytes were collected from superovulated female rats previously mated with males. sgRNA and Cas9 were microinjected into the male pronucleus. Injected zygotes were cultivated overnight to the two-cell stage to assess sgRNA toxicity. The resulting two-cell embryos were reimplanted into pseudopregnant foster mothers at 0.5 days post-coitum. To identify Knock-out animals, the targeted locus was amplified and sequenced. Founders harbouring base pair insertions or deletions (indels) in the *FGF21* coding region were identified and bred to generate heterozygous animals. The heterozygous lines were then maintained by breeding with Wistar wild-type animals.

All in vivo experiments were performed in male rats aged around 6 months (chow, *n* = 16, 8 per genotype; GAN, *n* = 32, 16 per genotype). Circulating FGF21 levels were absent, confirming the knockout.

### Plasma and liver biochemistry

Animals were fasted for 4 h in their home cage, after which blood was sampled from the sublingual vein, kept on ice, and centrifuged (5 min, 4 °C, 6000 × *g*) to generate EDTA-stabilised plasma. Insulin, alanine aminotransferase (ALT), aspartate aminotransferase (AST), triglycerides (TG), total cholesterol (TC), and liver TG and TC were determined as described previously^14^. Plasma glucose was measured by glucometer (Roche, Basel, Switzerland) in animals following a brief 4 h fast. FGF21 was measured in duplicate in EDTA plasma samples using the Mouse/Rat FGF21 Quantikine ELISA Kit (Bio-Techne, MF2100) in accordance with the manufacturer’s instructions. For alcohol preference studies, ALT was measured using the Rat ALT ELISA Kit (Abcam, Cambridge, UK) as per the manufacturer’s instructions.

### Liver histology

A liver biopsy section ( ≈ 200 mg) was dissected from the left lateral lobe at termination, fixed overnight in 4% paraformaldehyde, paraffin-embedded, and sectioned (3 µm thickness). Liver sections were stained with hematoxylin-eosin (HE), picro-Sirius Red (PSR; Sigma-Aldrich, Brøndby, Denmark), anti-galectin-3 (cat. 125402, Biolegend, San Diego, CA), alpha-smooth muscle actin (α-SMA, cat. Ab124964; Abcam, Cambridge, UK), or anti-type I collagen (Col1a1, cat. 1310-01, Southern Biotech, Birmingham, AL) according to standard procedures^14,15^.

### Automated deep-learning-based image analysis

Automated deep learning-based image analysis GHOST (Gubra Histopathological Objective Scoring Technique)^16^ was used for objective histopathological scoring of steatosis, lobular inflammation, hepatocyte ballooning, and fibrosis staging according to the MASH Clinical Research Network (CRN) scoring system^17^. GHOST was further used to obtain histopathological scoring-associated variables, including fraction of lipid-laden hepatocytes (%), number of inflammatory foci (foci/mm^2^), and percent proportionate area of fibrosis. Quantitative histology was performed using Visiomorph digital imaging software (Visiopharm, Hørsholm, Denmark) for the determination of liver fat (HE-staining), fibrosis (PSR, Col1a1), inflammation (galectin-3), and alpha-smooth muscle actin (α-SMA) expressed relative (%) to total sectional area. The percent area of positive staining was multiplied by the corresponding total liver weight to give an estimate of total liver marker content (mg).

### Liver RNASeq

Total RNA was isolated using RNeasy Minikit (Qiagen) according to the manufacturer’s protocol. Total RNA sequencing was performed by the Single-Cell Omics platform at the Novo Nordisk Foundation Centre for Basic Metabolic Research. Libraries were prepared using Universal Plus Total RNAseq with the NuQuant kit (Tecan) as recommended by the manufacturer. In brief, 250 ng total RNA was fragmented, followed by cDNA synthesis. Adaptors were ligated, and after stand selection, the cDNA was depleted from rRNA using AnyDeplete probes (Tecan). Following the final bead clean-up, libraries were quantified with NuQuant, quality checked using a TapeStation instrument (Agilent Technologies), and subjected to 52-bp paired-end sequencing on a NovaSeq 6000 (Illumina). Differential gene expression analysis was conducted using the DESeq2 R-package. Gene set analysis was performed using the PIANO version 1.18.1 R-package and the Stouffer method. The Reactome pathway database^18^ was used for gene annotation for gene set analysis. The Benjamini−Hochberg method (5% false discovery rate, FDR < 0.05) was used for multiple testing correction of *P*-values.

### Statistics

**Fig. 2:**
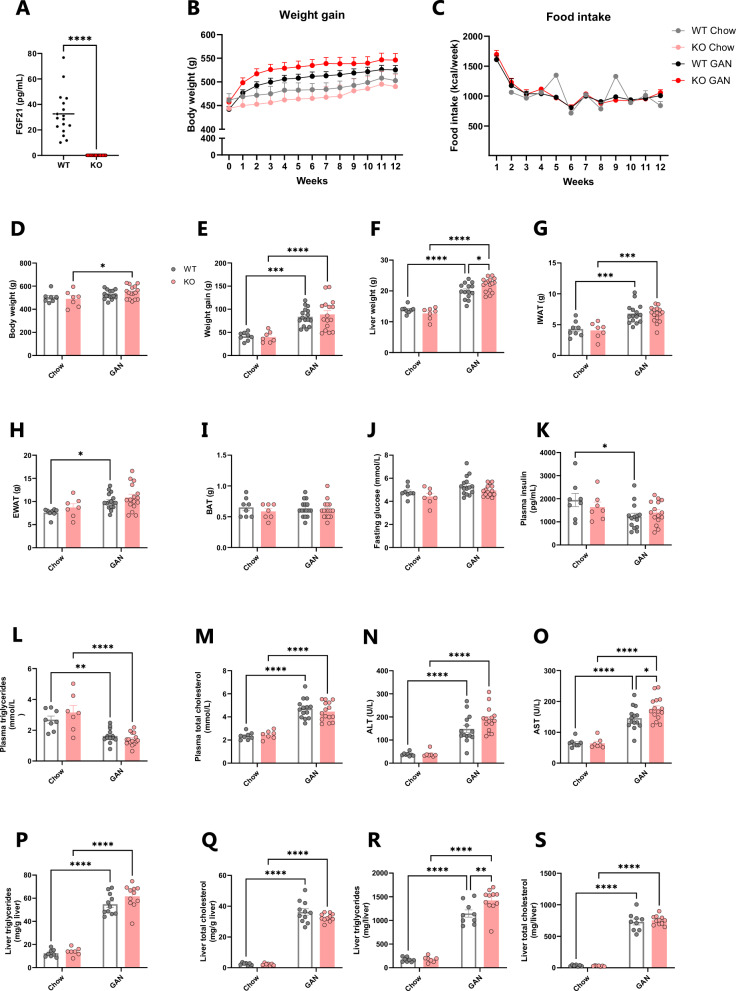
Effect of 12 weeks of GAN diet feeding on body weight, food intake, fat mass, metabolic and biochemical parameters in FGF21 KO rats. **A** Validation of circulating FGF21. **B** Body weight trajectories. **C** Food intake. **D** Terminal body weight. **E** 12-week body weight gain. **F** Terminal liver weight. **G** IWAT weight. **H** EWAT weight. **I** BAT weight. **J** 4 h fasted blood glucose. **K** 4 h fasted plasma insulin. **L** 4 h fasted plasma triglycerides. **M** 4 h fasted plasma total cholesterol. **N** 4 h fasted plasma ALT. **O** 4 h fasted plasma AST. **P** Liver triglyceriders. **Q** Liver total cholesterol. *n* = 7–16 rats/group, *P < 0.05, **P < 0.01, ***P < 0.001, ****P < 0.0001 (**A** unpaired *t*-test; **B**, **C** two-way repeated measures ANOVA; **D**–**S** two-way ANOVA). GAN Gubra-Amylin MASH, WAT white adipose tissue, IWAT inguinal WAT, EWAT epididymal WAT, BAT brown adipose tissue, ALT alanine transaminase, AST aspartate transaminase.

Except for RNA sequencing and deep learning-based image analysis, data were analysed using GraphPad Prism v9.0.2 software (GraphPad, La Jolla, CA). Data are presented as mean ± standard error of mean (SEM). Ordinary one-way or two-way ANOVAs were used for all statistical comparisons (specified in figure legend), except for histological scoring (Fig. 2A–E), where Mann–Whitney *U*-test with Bonferroni correction was used. A *P*-value of <0.05 was considered statistically significant.

## Results

### FGF21 deficiency increases liver weight and plasma transaminases but not body weight or fat mass

FGF21 activity was abrogated using the CRISPR/Cas9 technology to insert an artificial stop codon in exon 1, resulting in unstable mRNA and the replacement of eight amino acids critical for FGF21 activity (Fig. 1B, C and Supplementary Fig. 4). Accordingly, FGF21KO rats were deficient in circulating FGF21 (Fig. 2A). Histological and transcriptional markers of MASH were evaluated in WT and FGF21 KO rats after 12 weeks of GAN diet and compared to WT and FGF21 KO rats fed chow diet for 12 weeks (see study outline in Fig. 1). At baseline, prior to GAN diet induction, levels of plasma biochemical markers (insulin, triglycerides, total cholesterol, ALT and AST) were similar between all groups (Supplementary Fig. 2A–E). The GAN diet induced a transient increase in food intake (kcal/week) during the first one to two weeks, after which, food intake remained stable and similar between groups (Fig. 2C). GAN diet feeding for 12 weeks or FGF21 deficiency did not lead to changes in terminal body weight (Fig. 2D) but did increase body weight gain (Fig. 2E). GAN diet increased hepatomegaly which was further exacerbated by FGF21 deficiency (Fig. 2F). Furthermore, iWAT and eWAT mass was increased in response to GAN diet but not affected by FGF21 deficiency (Fig. 2G–I). 4 h fasted blood glucose levels were similar between all groups at week 11 (Fig. 2J), while 4 h fasted plasma insulin levels were only lower in WT GAN group compared to WT chow (Fig. 2K). Terminal biochemistry analysis displayed a GAN-driven increase in total plasma cholesterol, ALT, AST, and a decrease in plasma triglycerides (Fig. 2L–O). FGF21 deficiency in the context of GAN diet feeding further increased plasma AST and trended towards increasing plasma ALT (*p* = 0.05). GAN-diet feeding increased total levels of liver triglycerides and total cholesterol in both WT and FGF21 KO rats (Fig. 2R, S), but not when adjusted for liver weight (Fig. 2P, Q).

### FGF21 deficiency does not worsen GAN-driven hallmarks of MASH

**Fig. 3:**
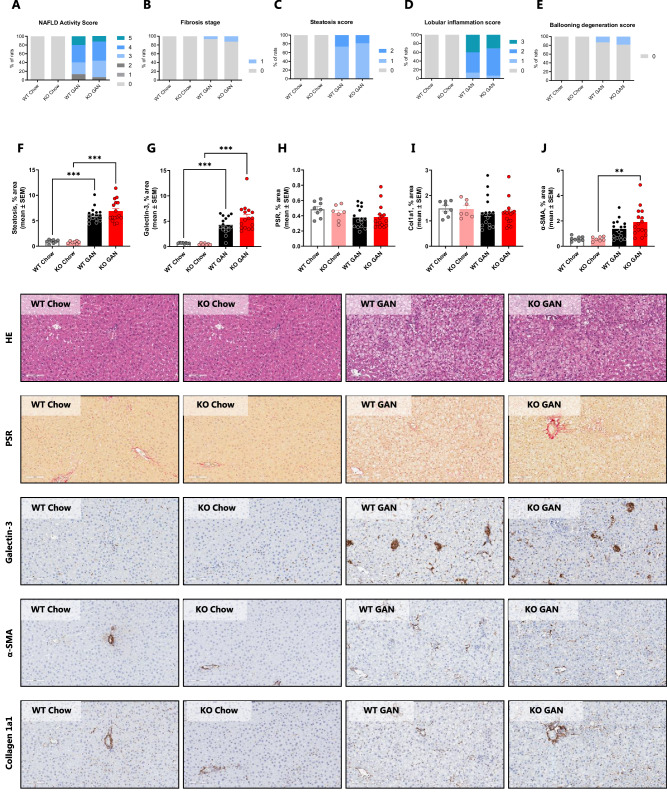
Effect of 12 weeks of GAN diet feeding and FGF21 deficiency on MASLD Activity Score, fibrosis stage, and quantitative histological markers. Histopathological scores were determined by the Gubra Histopathological Objective Scoring Technique (GHOST), a deep learning-based image analysis. **A** NAS. **B** Fibrosis stage. **C** Steatosis score. **D** Lobular inflammation score. **E** Ballooning degeneration score. No differences at significance level 0.05 (WT Chow vs KO Chow and WT GAN vs. KO GAN, Mann–Whitney *U*-test with Bonferroni correction). Percent area of **F** steatosis, **G** Galectin-3, **H** PSR, **I** Collagen 1a1, and **J** α-SMA. *n* = 7–16 rats/group, *P < 0.05, **P < 0.01, ***P < 0.001 (two-way ANOVA). MASLD metabolic dysfunction-associated fatty liver disease, GAN Gubra-Amylin MASH, NAS NAFLD activity score, GHOST Gubra histopathological objective scoring technique, PSR picrosirius red, α-SMA alpha-smooth muscle actin.

Rats fed the GAN diet for 12 weeks exhibited moderate MASLD (NAS of 2–5), with more than 56% of the animals featuring with NAS of ≥4 (Fig. 3A). Changes in NAS were primarily driven by increases in steatosis and lobular inflammation score (Fig. 3C, D). There was no difference between WT and FGF21 KO rats. In both groups, less than 20% of animals presented ballooning degeneration in response to GAN feeding (Fig. 3E). In support of changes in histopathological scores, quantitative markers of steatosis (% area of steatosis, Fig. 3F) and inflammation (% area of galectin-3, Fig. 3G) were increased in response to GAN feeding, but not affected by genotype status. 12 weeks of GAN diet feeding did not induce liver fibrosis as evident from fibrosis staging and corroborated by quantitative histological markers of fibrosis (PSR % area, Fig. 3H; Col1a1% area, Fig. 3I). However, α­SMA, marker of hepatic stellate cell activation, was increased in the KO GAN group compared to KO Chow (Fig. 3J).

### Minimal changes in liver transcriptome signatures

**Fig. 4:**
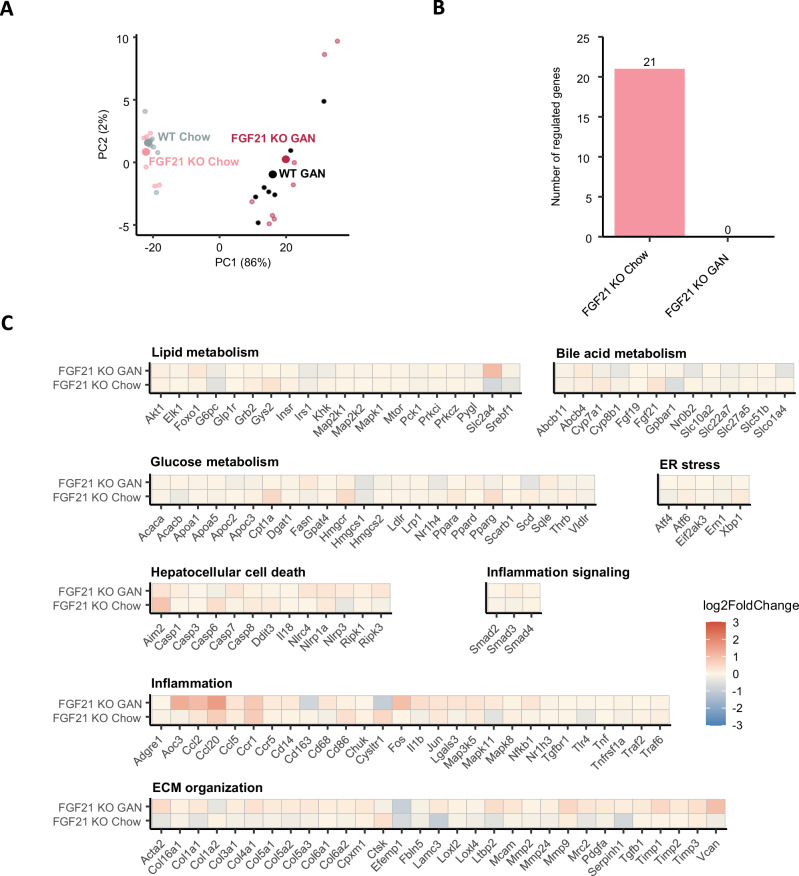
Transcriptional alterations in response to GAN diet feeding and FGF21 deficiency. **A** Principal component analysis (PCA) of samples based on the top 500 most variable gene expression levels. **B** Total number of genes differentially expressed versus WT Chow and WT GAN, respectively. **C** Heatmaps displaying regulations in MASH and fibrosis-associated candidate genes (log2-fold change compared with respective WT group). Blue colour gradients indicate downregulation of gene expression, red colour indicates upregulation of gene expression. *n* = 7–8 rats/group. GAN, Gubra-Amylin MASH.

A principal component analysis (PCA) of the 500 most variable genes displays clustering based on diet type along the PC1 axis, accounting for 86% of the variance (Fig. 4A). Genotype status had limited effect on the number of differentially expressed genes (DEGs) (Fig. 4B). The FGF21 KO chow group showed 21 regulated genes compared to WT chow, while there were no DEGs between FGF21 KO GAN and WT GAN. No MASH-associated candidate genes were significantly regulated, but FGF21 status conferred minor trends in global gene expression (Fig. 4C).

### Endogenous FGF21 does not regulate alcohol preference or binge drinking in rats

Finally, we explored the role of FGF21 to regulate behaviour in the rat by studying its role in alcohol preference and in binge-drinking paradigms in separate cohorts. However, rats lacking endogenous FGF21 did not exhibit increased preference for alcohol during continuous access to either water or 10% ethanol (Supplementary Fig. 3A) and did not show increased alcohol consumption during two separate binge-drinking paradigms (Supplementary Fig. 3B–E). Despite this, FGF21 KO rats exhibited a significant increase in ALT during this period (Supplementary Fig. 3F).

## Discussion

MASH is an advanced stage liver disease with no FDA-approved treatments. It is evident that the liver hormone FGF21 plays a role in both the development, and treatment of MASH with FGF21 analogues demonstrating positive results in clinical trials. However, the mechanisms underlying the role of FGF21 in liver disease remain unclear.

Here, we capitalised on the advancements of the gene-editing technology Crispr/Cas9, and the potential utility of rat models, by generating a novel FGF21 knockout rat model which could recapitulate the hallmarks of human disease when combined with the GAN diet. We demonstrate that FGF21 deficiency in the context of GAN diet feeding exacerbated hepatomegaly and plasma transaminases. However, both WT and FGF21 knockout rats display comparable weight curves and excess fat mass on a GAN diet, with no difference in FGF21 knockout animals. GAN diet feeding increased MASLD-associated plasma and liver markers (plasma TC/ALT/AST, liver TG/TC), with an additional worsening of plasma ALT/AST exerted by FGF21 deficiency. GAN diet consistently drove a liver phenotype characterised by increased liver steatosis and inflammation, evident from histopathological scores (NAS, steatosis score, lobular inflammation score) and supported by quantitative image analysis (steatosis% % area, Galectin-3). There was no difference between WT and KO for any of the histological endpoints. In concert, transcriptional gene signatures clearly clustered according to diet, with no additional effect of genotype status. However, the GAN diet was not sufficient to drive advanced liver pathology with fibrosis.

Whilst mice are commonly used models to study MASLD and MASH, primarily due to the genetic toolkits available, fibrosis models were originally developed in rats as they develop more robust fibrosis and are more susceptible to the histological hallmarks of MASH in response to diet^19,20^. FGF21 knockout mice on a high-fat diet exhibit a worsening of metabolic liver injury, hepatic steatosis, and MASH-HCC transition^21,22^. Here, we describe a mildly exacerbated liver phenotype compared to wild-type animals, however, without displaying a worsening on histopathological endpoints. Although FGF21 analogues consistently reverse or reduce MASH in mouse models and FGF21 reduces body weight^23^ and lowers blood glucose in rats^8,24^, endogenous FGF21 in the current study design is not sufficient to drive the full-blown MASH with fibrosis.

The reasons for this are not clear, though it could be speculated that extending the diet-induction period could give rise to liver fibrosis. Indeed, mice develop fibrosing MASH after ≥28 weeks of GAN diet feeding^16,25^. As such, it is possible that 12 weeks was insufficient time for rats to develop an advanced liver phenotype. Although, as discussed above, FGF21 KO mice exhibit exacerbated liver phenotypes in response to multiple different diets, it would have been expected, based on current evidence, that an FGF21-deficient rat would develop exacerbated liver phenotypes even over 12 weeks of exposure to the GAN diet. Similarly, it is possible that utilising younger animals may have yielded a more pronounced phenotype. Typically, mouse and rat models of diet-induced obesity commence between 6–10 weeks of age, around adolescence, as this is considered optimal in terms of weight gain, and often produces an experimentally clear metabolic phenotype in as little as 8–10 weeks. Given our aim was to develop a translationally relevant model that mimicked the hallmarks of human MASH, we commenced a MASH-inducing GAN diet in 24-week-old adult rats, which, comparatively, will exhibit a lesser capacity for weight gain and therefore metabolic deterioration. However, it could be argued that, if endogenous FGF21 was an important regulator of liver pathology in rats, the age at which animals commenced a diet should be less decisive. Indeed, FGF21 KO mice exhibit a subtle, but significant increase in body weight by 24 weeks on standard chow^3^, which was not evident here and which one would expect to be exacerbated during high-fat feeding.

Finally, we show that endogenous FGF21 does not regulate drinking behaviour in rats. This is despite a wealth of evidence implicating the beta-klotho-FGF21 pathway in the genetics of alcohol-use disorder^26,27^, demonstrating that FGF21 KO mice (global, liver and CNS-specific) drink more ethanol in both two-bottle preference and binge-drinking paradigms, whilst FGF21 treatment reduces drinking in mice and non-human primates^13,26^. Again, why endogenous FGF21 does not regulate drinking in the rat is unclear. There are strain-specific differences in alcohol intake and susceptibility to ‘addiction’, but the Wistar rat, which we used as background, is commonly used due to their high alcohol preference and will choose alcohol over social interaction^28^. Importantly, the Wistar strain is also the most susceptible to MASH^29^ and thus represents the best background strain to investigate the role of FGF21 in rats.

In conclusion, through in-depth molecular, histological and behavioural characterisation we demonstrate that endogenous FGF21 mildly exacerbates hepatomegaly and biochemical markers of hepatic dysfunction in response to GAN diet and alcohol in rats.

## Supplementary information

Supplementary Figures

## Data Availability

Data will be made available upon reasonable request.
