# Supplementary material for: FGF21 deletion mildly exacerbates hepatic dysfunction in GAN diet and alcohol fed rats
Source: NPJ Metab Health Dis. 2025 May 28;3:21. doi: 10.1038/s44324-025-00062-5 (PMC12441114; doi:10.1038/s44324-025-00062-5)
Supplement: Supplementary file 1 — Supplementary Figures [file 44324_2025_62_MOESM1_ESM.pdf]

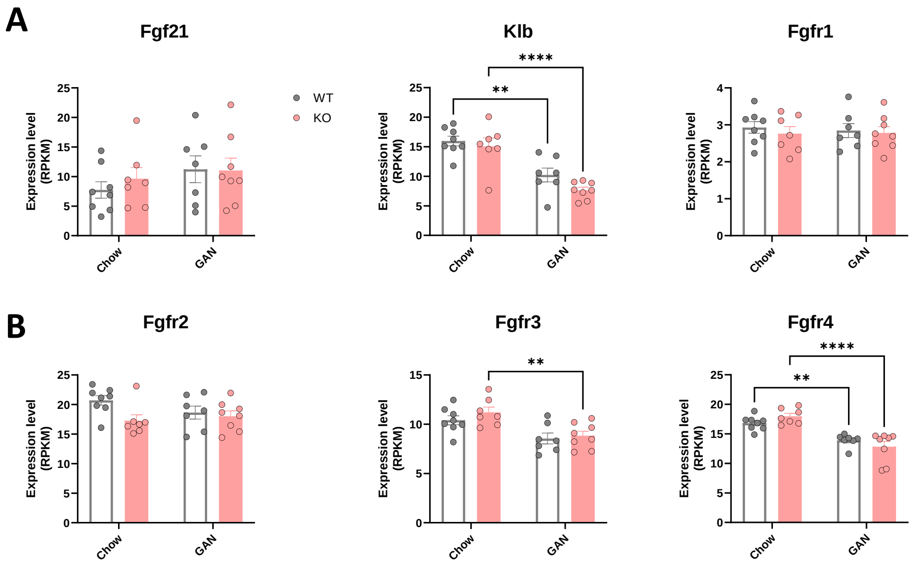

Supplementary figure 1.

Hepatic mRNA expression of fibroblast growth factor 21 (Fgf21), b-klotho (Klb), FGF receptor 1 (Fgfr1), FGF receptor 2 (Fgfr2), FGF receptor 3 (Fgfr3), and FGF receptor 4 (Fgfr4). **P < 0.01, ***P < 0.001 (Two-way ANOVA). n =7–8 rats/group.

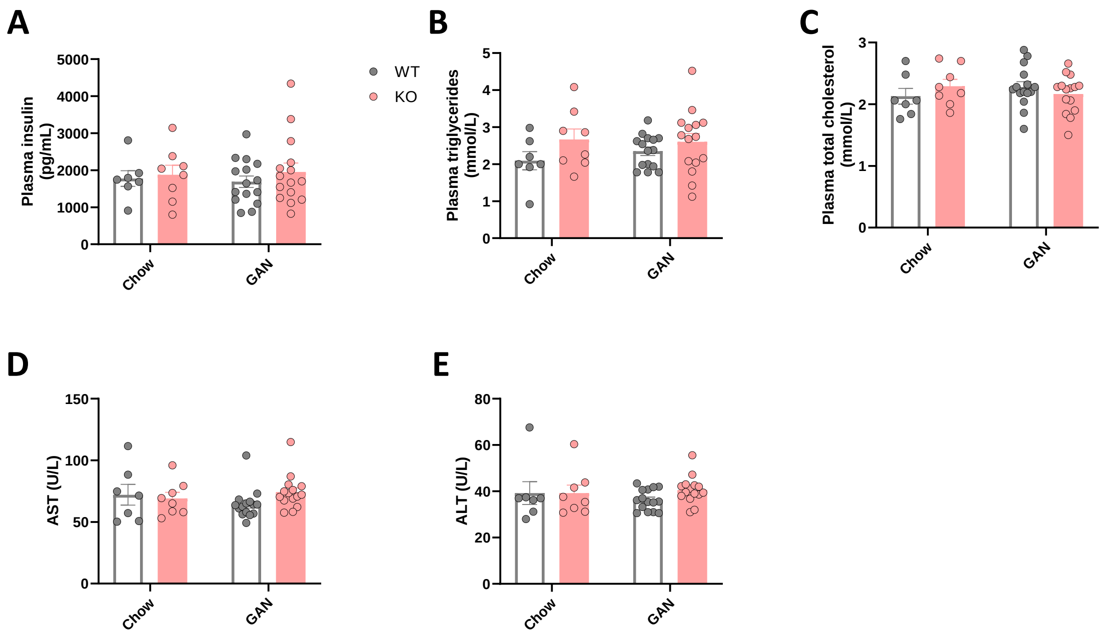

Supplementary figure 2.

Baseline levels, prior to GAN diet induction, of plasma biochemical markers, 4h fasted. **(A)** Plasma insulin. **(B)** Plasma triglycerides. **(C)** Plasma total cholesterol. **(D)** Plasma ALT. **(E)** Plasma AST. n =7–16 rats/group, P > 0.05 (Two-way ANOVA).

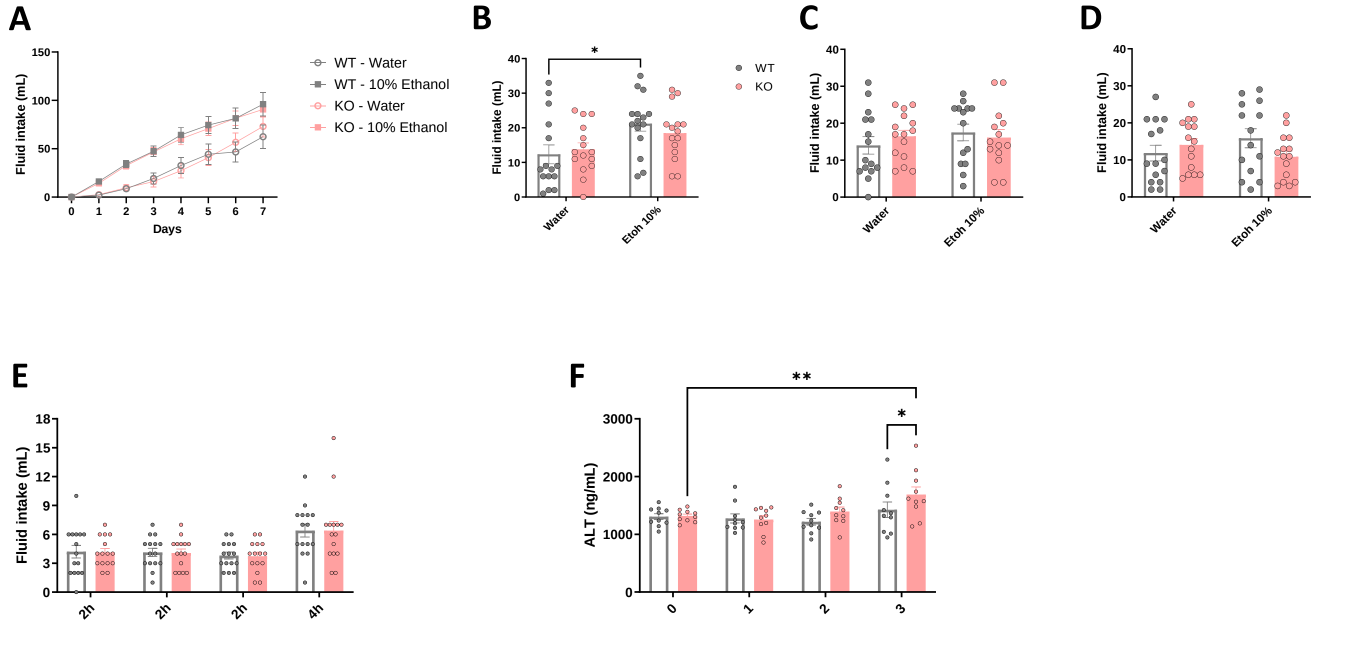

Supplementary figure 3.
Drinking behaviour of FGF21 KO rats during (A) continous alcohol preference (B-D) Intermittent Access Two Bottle Choice days 1, 2 and 3 and (E) Drinking in the Dark studies. (F) Weekly changes in circulating ALT. *P < 0.05, **P < 0.01 (A, E & F, Two-way repeated measures ANOVA; B-D, Two-way ANOVA). n =15 rats/group.

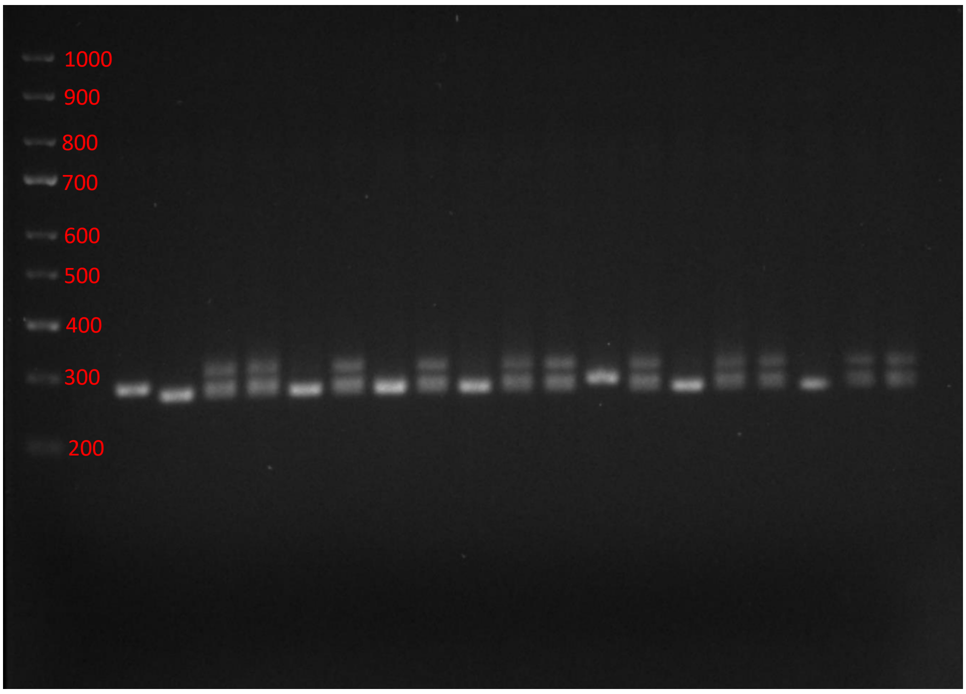

Supplementary figure 4.

The original gel image for genotyping in Fig. 1
